# Motion and Distribution of Floating Grain in Direct-Chill Casting of Aluminum Alloys: Experiments and Numerical Modeling

**DOI:** 10.3390/ma13235379

**Published:** 2020-11-26

**Authors:** Qipeng Dong, Yanbin Yin, Zhen Zhu, Hiromi Nagaumi

**Affiliations:** 1 High-Performance Metal Structural Materials Research Institute, School of Iron and Steel, Soochow University, Suzhou 215021, China; zhuzhen6821@163.com; 2 State Key Laboratory of Advanced Metallurgy, University of Science and Technology Beijing, Beijing 100083, China; ustbwenwu@sina.com

**Keywords:** distribution, floating grain, direct-chill casting, aluminum alloys, simulation

## Abstract

Sedimentation of free-floating grains is the main origin of the negative centerline segregation in direct-chill casting of aluminum alloys. This study examines the motion and distribution of the floating grains during casting using experimental measurements and numerical modeling. The typical floating grains consisting of interior solute-lean coarse dendrites and periphery fine dendrites were experimentally observed only in the central region of the billet along with the negative segregation. The billet exhibits the strongest segregation at the center where the most floating grains are found. In simulations, under the action of the convection and the underlying forces, the grains floating in the transition region exhibit different motion behaviors, i.e., settling to the mushy zone, floating in the slurry zone, and moving upward to the liquid zone. However, most grains were transported to the central region of the billet and then were captured by the mushy zone and settled. Therefore, the floating grains comprise the largest share of the grain structure at the center of the billet, in agreement with the experimental results. Moreover, the simulation results indicate that the increased size of the grains promotes the sedimentation of the floating grains. These results are important for the future alleviation of negative centerline segregation in direct-chill casting of aluminum alloys.

## 1. Introduction

Macrosegregation is a thorny problem that is commonly found in large-scale castings and ingots and cannot be eliminated during downstream heat treatment [1,2,3,4]. Negative centerline segregation typically occurs in the direct-chill (DC) casting of aluminum alloys [4,5,6,7], and both the solidification shrinkage and sedimentation of free-floating grains have been shown to result in the unusual segregation patterns. It is known that the solidification shrinkage in DC casting of aluminum alloys is an intrinsic phenomenon that always occurs in the solidifying mushy zone whereas the accumulation of floating grains at the billet center depends on the casting condition [8]. This indicates that the centerline segregation of DC casting can be alleviated by minimizing the contribution of floating grains. Therefore, it is necessary to obtain a deep understanding of the macrosegregation mechanism induced by floating grains.

The experimental observation of a duplex microstructure [9,10], i.e., a mixture of the coarse- and fine-DAS (secondary dendrite arm spacing) dendrites has been considered to be evidence of the floating grains. It is typically assumed that the coarse dendrites are solute-lean, and their presence and accumulation at the billet center result in negative segregation (noted that the eutectic elements are discussed herein) [7,10,11]. Therefore, the grains with the coarse-DAS and more solute-lean dendrites are the floating grains. During DC casting of aluminum alloys, many factors may affect the occurrence of the floating grains such as the alloys composition, size of the ingot, grain refinement, and solidification conditions. However, the distribution of the floating grains must play a key role for the negative centerline segregation. Turchin et al. [12] carried out an investigation on the solidification of an Al-4.5pct Cu alloy under forced-flow conditions in a shallow cavity by experiments and numerical simulations. Their results clearly revealed the presence of an interaction between the convective flow and the grain structure evolution during solidification, including the formation of peculiar grains and dendrite morphology. Recently, the presence of the free-floating grains in the transition region of a solidifying DC-cast ingot was experimentally verified by Joseph [13] who found that the quenched microstructure consists of both large grains (already present in the two-phase region prior to sampling) and fine crystals (solidified in the quenching mold). In addition, the free-floating grains were considered to originate from the heterogeneous nucleation or detached dendrites in DC casting [7,14,15,16,17,18]. This explains the more severe centerline segregation often observed for grain-refined alloys compared to that of the non-grain refined alloys [10,11,19]. For grain-refined alloys, more grains freely float in the transition region of casting because of the increased nucleation on the grain refiner. Moreover, a novel jet mixing method was proposed by Wagstaff and Allanore [20,21], and the resulting turbulent jet was believed to suspend the settled grains at the sump bottom, and thus reduce the negative centerline segregation. This work suggests that the centerline segregation in DC casting of aluminum alloys can be alleviated by preventing the accumulation of the free-floating grains.

Knowledge of the origin of the sedimentation and accumulation of the floating grains at the central region of the DC casting is critical for obtaining a thorough understanding of the macrosegregation mechanism induced by floating grains. Unfortunately, the results of all of the above-mentioned experimental studies did not reveal that because the DC casting of aluminum alloys is known to be a highly complex process at high temperature. Therefore, numerical modeling that can visualize the ongoing transport process has been widely applied in the research on macrosegregation in DC casting [6,22,23,24,25,26,27,28,29]. Nevertheless, despite the many numerical studies that have successfully included the influence of floating grains in the macrosegregation simulation with analytical modeling [23,26,27,28,29,30], the motion and distribution of the free-floating grains in the transition region of DC casting are still unclear.

Hence in the present study, the segregation and grain structure of a commercial DC-cast billet of Al-Mg-Si alloys were examined experimentally. The floating grains in the billet were identified according to the microstructure and microsegregation analysis, and the distribution of the floating grains in the DC-cast billet was experimentally determined. In addition, the motion and distribution of the floating grains in the transition region of a solidifying DC casting were numerically studied with an Euler-Lagrange coupled model. The influence of the size of the grains on their distribution in a solidified billet was investigated. Based on these results, the origin of the experimentally observed distribution of the floating grains was elucidated. The results will deepen our understanding on the macrosegregation mechanism induced by the sedimentation of free-floating grains, and will also contribute to the alleviation of the negative centerline segregation in DC casting by preventing the accumulation of the floating grains at the casting center.

## 2. Experimental

Experiments were carried out with a commercial Al-Mg-Si (Al-1.1Mg-1.0Si-0.44Cu-0.48Mn-0.3Cr (wt.%)) DC-cast billet with 152 mm in diameter (provided by China Hongqiao Group Limited, Binzhou, China). Four samples were cut along the radial direction for the composition analysis with a spark spectrum analyzer (Spectro, Kleve, Germany), and the average values were given. The interval between the two adjacent measurements was approximately 10 mm. For the macrostructure investigation, the cross-section of the billet was etched using a 10% NaOH solution and a Nital cleaning agent after milling. Microstructural examination was carried out on four metallographic specimens (20 × 20 mm in size) taken at different distances away from billet center, i.e., 0 mm, 20 mm, 40 mm, 60 mm, after electrolytic etching at 20 V DC in Barker’s reagent, consisting of 5 mL HBF4 (48%) in 200 mL H_2_O. Noted to exclude the influence of surface segregation, the analysis of the sample at the surface referred to in the subsequent discussion was initiated at a distance of ~10 mm from the actual surface of billet. The specimens were re-polished and etched for 8 s at 0 °C with Weck’s reagent [31,32] to reveal the dendrite structure and floating grain further. For the microsegregation study, EPMA (electron probe micro-analyzer, Shimadzu EPMA-1720, Kyoto, Japan) area-scanning, operating at an acceleration voltage of 15 kV, electron beam current of 100 nA and sampling time of 100 ms, was performed on selected grains. On basis of the microstructure and microsegregation results, floating grains can be determined. The fractions of the floating grains in grain structure were measured on photographs using the random line intercept method.

## 3. Numerical Modeling

### 3.1. Model Description

A two-dimension model developed based on the continuum model [33,34] was used to calculate the fluid flow, heat transfer, and solidification process during DC casting of aluminum alloys. The motion and distribution of the grains (represented by particles) in the casting were simulated using a particle transport model [35,36] based on the Lagrangian approach. The conservation equations of the model applied here are listed in Table 1. The following assumptions were made in the simulations.

(a) The molten aluminum was considered as an incompressible Newtonian fluid.
(b) Local thermodynamic equilibrium was assumed at the solid–liquid interface
(c) The shrinkage-induced flow which mainly acts in mushy zone was ignored.
(d) The influence of the grains morphology on the motion behavior was neglected, i.e., the grains were treated as the spherical particles. This assumption is relatively reasonable for the DC casting of aluminum alloys, because grain refining (Al-Ti-B master alloys are most widely used as inoculants) is commonly employed in the industrial production of aluminum alloys, thereby the grain morphology of the resultant billets or ingots is typically equiaxed [8].
(e) Herein the growth of the grains in the transition region of the DC casting are not considered yet.

### 3.2. Numerical Procedure

The simulations were carried out with an open-source software OpenFOAM (OpenFoam240) based on the finite volume method. The PIMPLE transient solver (a merged PISO-SIMPLE algorithm) was applied to solve the pressure-velocity coupling. The calculation consists of two parts, and the transient simulation of the fluid flow and solidification provides an initial condition for the subsequent modeling of particles motion. During simulation, the molten aluminum alloy with the casting temperature enters into the billet through the inlet at the top. The inlet velocity profile was assumed to be flat and determined based on the inlet-outlet mass balance. The solidified billet leaves the calculation domain from the outlet with the casting speed. 

In the simulation, the billet surface was treated as a moving wall, and the velocity was set to equal casting speed. The thermal boundary conditions were given based on the Fourier condition:

(1) $$q_{s}=h(T_{surf}-T_{e})$$

where *T_surf_* is the surface temperature of billet, *T_e_* is the environment temperature, and *h* is the heat transfer coefficient.

For the primary cooling (mold zone), the heat transfer coefficient was treated as a function of solid fraction to consider the influence of air gap:

(2) $$h=h_{contact}(1-f_{s})+h_{air}\cdot f_{s}$$

Heat transfer coefficient in the secondary cooling region varies with the surface temperature, and the values can be referred to the previous work [37], wherein the boundary condition had been experimentally validated.

The operation parameters and thermo-physical parameters applied in the simulation are listed in Table 2.

**Figure 1:**
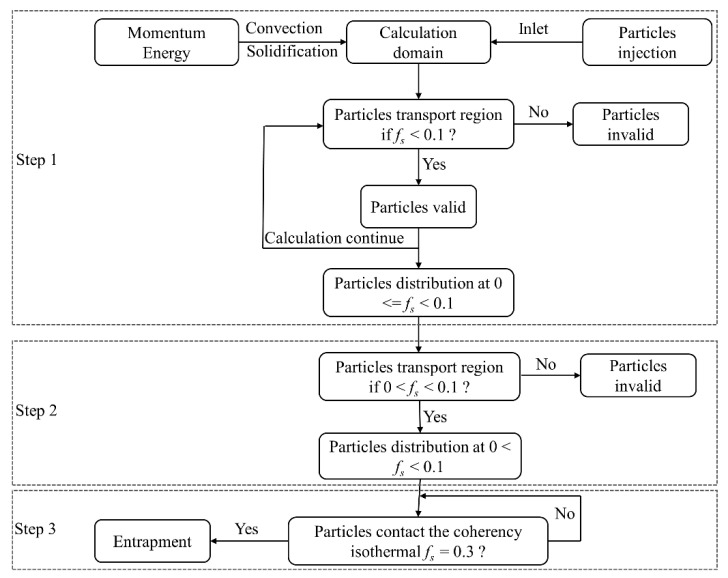
Flow chart of the particles motion and entrapment simulation.

**Figure 2:**
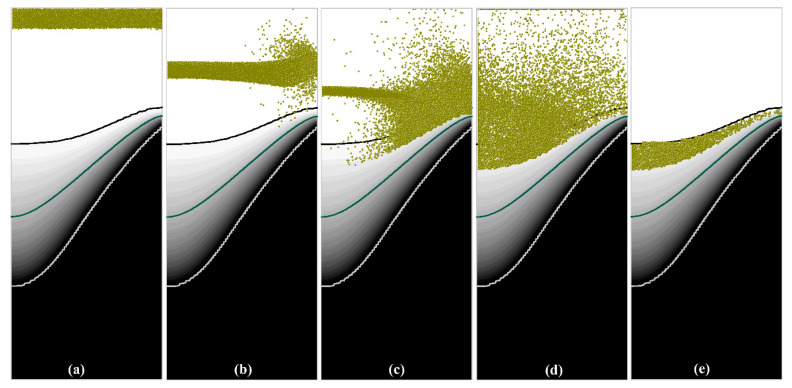
Numerical procedure for obtaining the initial distribution of the particles in DC casting, (**a**) particles injection, (**b**–**d**) motion and distribution of the particles at different calculation times, (**e**) particles distribution within 0 < *f_s_* < 0.1.

As mentioned above, the free-floating grains in the transition region of casting are considered to originate from the nucleus or detached dendrites. However, implementing the generation of the grains in a macroscopic simulation is currently impractical. Therefore, in this study, the grains were assumed to be initially distributed in a region adjacent to the liquidus, i.e., solid fraction (fs) between 0 and 0.1. The initial distribution of the particles was numerically obtained, and the simulation can be divided into three steps, as depicted in Figure 1. And the simulated motion and distribution of the particles in the casting at different calculation procedures are shown in Figure 2. First, a number of particles were introduced into the casting from the inlet (shown in Figure 2a), but only the particles within the region of *f_s_* < 0.1 are retained, as shown in Figure 2b,c. The particles settled and were transported elsewhere in the casting after they were introduced. During this calculation, the particles that settle to the region of *f_s_* > 0.1 were deliberately deleted. Then the distribution of the particles in a limited region with solid fraction below 0.1 was obtained, as shown in Figure 2d. Based on that, the criterion for the particle’s validity was modified to be 0 < *f_s_* < 0.1. This implies that the particles in the liquid region would be deliberately deleted during the simulation. After a short calculation, only the particles within 0 < *f_s_* < 0.1 were retained, as shown in Figure 2e. Third, the motion and entrapment of the particles in DC casting were simulated. Herein, the entrapment criterion for the particles was set based on the solid fraction, i.e., the particles will be entrapped if they contacted the coherency isothermal (*f_s_* = 0.3).

## 4. Results and Discussion

### 4.1. Experimental Examination

**Figure 3:**
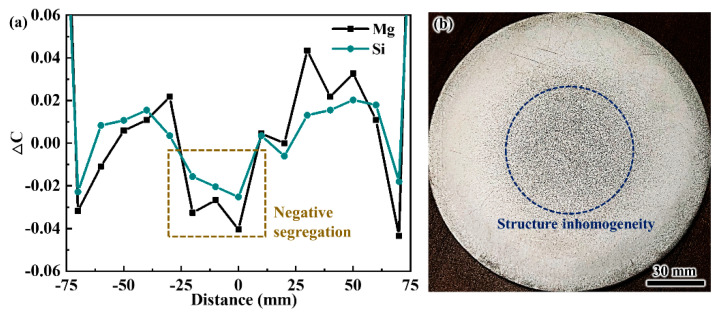
(**a**) Macrosegregation profiles of solute Mg and Si, (**b**) macrograph of the billet cross-section.

Figure 3a illustrates the macrosegregation profiles of solute Mg and Si across the billet cross-section. The degree of the macrosegregation in alloys casting is normally evaluated using relative composition deviation (also called degree of segregation in some works), Δ*C* = (*C_i_* − *C*_*i*,0_)/*C*_*i*,0_, where *C_i_* and *C*_*i*,0_ are the measured and nominal concentration of element *i*, respectively. A positive value of Δ*C* indicates the occurrence of positive segregation, and a value less than 0 represents negative segregation. It is observed that inverse segregation is typically observed in the DC-cast billet, i.e., positive segregation is found at the periphery of the billet while the center is solute lean. The positive surface segregation is known to be induced by the exudation of the solute-enriched liquid through the solidifying shell of a casting. By contrast, negative segregation for the centerline is quite complicated. In addition to the solidification shrinkage, the floating grains are also believed to contribute to the negative segregation because of the experimental observation of the duplex grain structure. As observed from the macrograph of the billet cross-section (shown in Figure 3b), the inhomogeneous macrostructure is clearly visible in the central region of the billet and its position corresponds precisely to that of the composition inhomogeneity.

**Figure 4:**
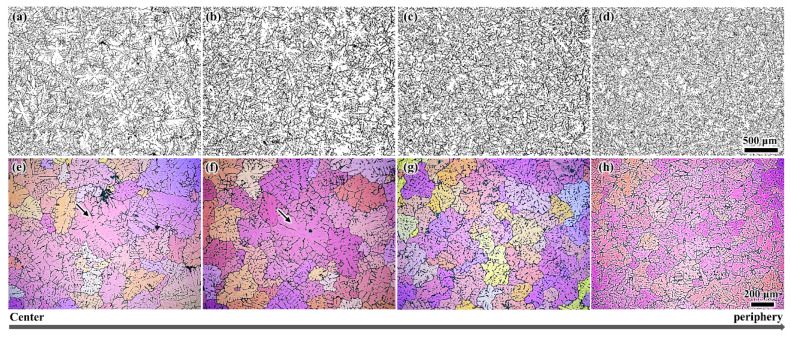
Typical microstructural results of the DC cast billet from center to the periphery for different distances from billet center (**a**) and (**e**) 0 mm, (**b**) and (**f**) 20 mm, (**c**) and (**g**) 40 mm, (**d**) and (**h**) 60 mm.

Figure 4 shows the typical microstructure results obtained from the four specimens with different distances from the billet center, as well as the polarized light microscopy observations of the grain structure. All of the grains are observed to be equiaxed. The grain structure at the billet periphery and nearby region is highly uniform, with an average size of approximately 180 μm. By contrast, some peculiar grains (identified with the arrows) with a size of greater than 400 μm can be clearly observed in the central region of the billet. However, even in the central region, the majority of the observed grains are regular, as shown in Figure 4a,b,e,f. The experimentally observed combination of the peculiar and regular grain structure should be the previously reported duplex microstructure [9,10], and the peculiar grains can be preliminary determined to be the floating grains based on the work of Eskin et al. [7,10,11]. To confirm this conclusion, further analysis of the microstructure and microsegregation was performed.

**Figure 5:**
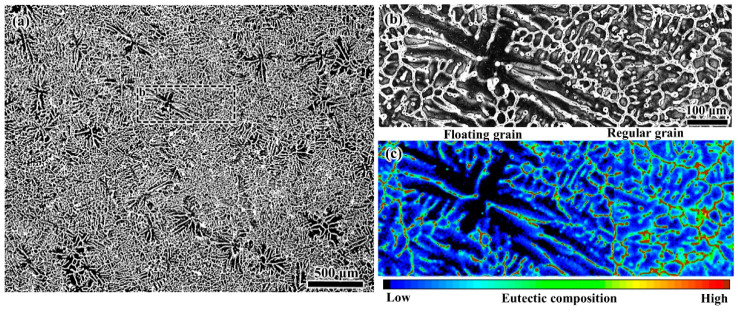
(**a**) Microstructure results at billet center after etching by Weck’s reagent, (**b**) typical duplex grain structure, (**c**) eutectic composition results obtained with EPMA.

The microstructure results shown in Figure 5a clearly present a duplex grain structure at the center of the DC-cast billet. Many coarse-DAS dendrites abruptly appear among the fine-DAS dendrites. A magnified view of the typical duplex grain structure is shown in Figure 5b, where the dendrites morphology can be identified. The corresponding results of the composition analysis obtained by EPMA are shown in Figure 5c. The DAS values of the coarse dendrites are nearly double that of the surrounding fine dendrites and the coarse-DAS dendrites are shown to contain lower concentrations of solutes than the fine dendrites. In addition, some fine-DAS dendrites appear to grow based on the coarse-DAS dendrites. This can reflect the solidification sequence of the duplex structure, i.e., the coarse dendrites must solidify prior to the fine dendrites, and this corresponds exactly to the mainstream floating mechanism. Some grains freely float and grow in the slurry zone, while the slurry zone of a DC casting has relatively lower temperature gradient, allowing the slow growth and coarsening of the dendrites. Then, the grains were transported to the central region of the billet and entrapped by the mushy zone where rapid solidification results in the formation of the surrounding fine-DAS dendrites. In addition, mushy zone is solute-enriched due to the microsegregation and solidification shrinkage, whereas the concentrations of solute elements in slurry zone are relatively lower. Therefore, the grains floating in the slurry zone are solidifying from liquid which has suffered little or no segregation and lower temperature gradient. This contributes to the formation of the coarse-DAS and more solute-lean dendrites of the floating grains. However, the fixed grains solidify from the solute-enriched liquid with higher temperature gradient, thereby the fixed grains possess the fine-DAS and less solute-lean dendrites. And the size of the floating grains is thus larger than that of the fixed grains in response to the different freezing conditions. The comparison in the microscale features between the fixed and floating grains can also be referred to the previous work [38]. Based on the microscopic features, the peculiar grains observed in the central region of the billet are confirmed to be the floating grains.

**Figure 6:**
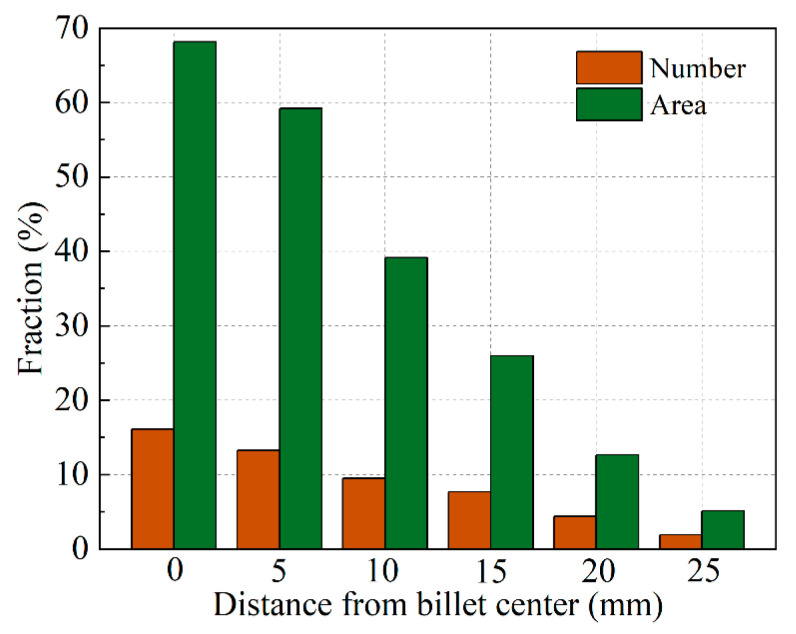
Experimental distribution of the floating grains in the central region of the DC-cast billet.

The composition results shown in Figure 3a display the greatest negative segregation at the center of the billet where more floating grains were found compared to the other regions, as shown in Figure 4. Therefore, based on the above analysis, the distribution of the floating grains in the DC-cast billet was experimentally investigated, and the statistical analysis results including the number fraction and area fraction are shown in Figure 6. Since the floating grains are only observed in the central region of the billet, the analysis was mainly carried out in a region of 0–30 mm away from billet center. The floating grains comprise the largest share of the grain structure at the billet center, with a number fraction of approximately 16% and area fraction of nearly 70%. Then, the floating grain number and area fractions gradually decrease to 1.9% and 5.1%, respectively, at a distance of 25 mm from the billet center. In fact, the dendrite structure that contributes to the negative segregation is not so much. This is because the floating grain itself consists of the interior coarse- and surrounding fine-DAS dendrites, while only the coarse-DAS dendrites contribute to negative segregation (as shown in Figure 5). For instance, the coarse-DAS dendrites only occupy ~28.95% of the floating grain shown in Figure 5. If we ignore the influence of the morphology variation of the floating grains, the coarse-DAS dendrites account for approximately 20.26% of the grain structure at the billet center. Considering the relatively low composition and the special distribution of the coarse-DAS dendrites, it is not surprising that they give rise to the negative centerline segregation of DC-cast billets. The degree of negative segregation gradually decreases with the increasing distance away from the billet center because of the decreased amount of floating grains.

### 4.2. Motion and Distribution of Floating Grains

**Figure 7:**
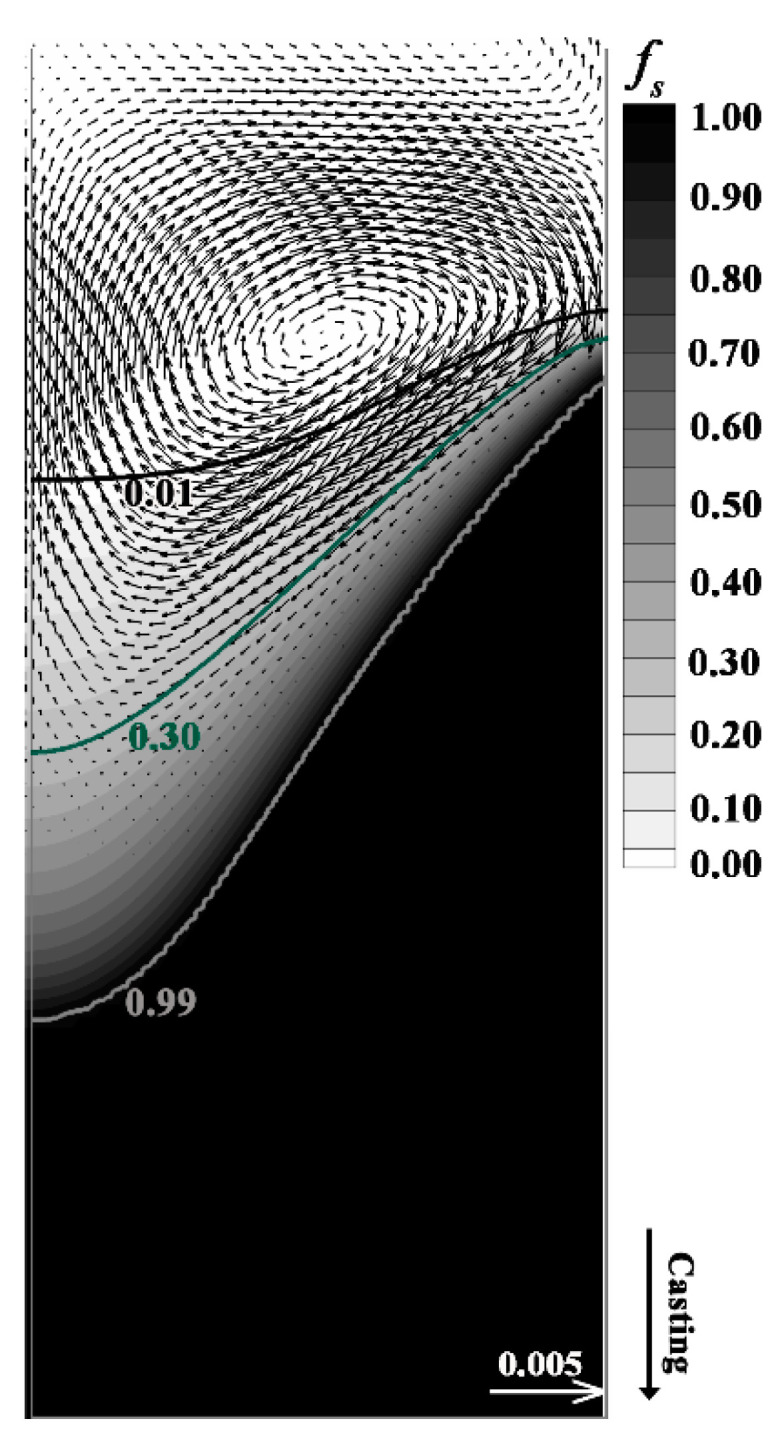
Simulated solid fraction and vector plot of the relative velocity (v-v_cast_) in DC casting.

To investigate the origin of the special distribution of the floating grains in the DC-cast billet, the motion and entrapment of the grains in DC casting were numerically modeled. The predicted results of the fluid flow and solidification that largely determine the motion and distribution of the floating grains are shown in Figure 7. The transition region of casting is divided into two parts, i.e., the slurry and mushy zones that are described by three characteristic isograms of solid fraction, i.e., 0.01, 0.99, and 0.30 (assumed coherency fraction). In DC casting of aluminum alloys, some grains originating from heterogeneous nucleation or detached dendrites [7,14,15,16,17,18] move and grow within the solidifying transition region, particularly in the slurry zone, where the grains can freely travel and thus will be transported elsewhere by the convection, while the grains that move into the mushy zone will be trapped. The results show that the slurry zone comprises approximately a half of the transition region, although it has a much smaller temperature range than the mushy zone (23 °C vs. 54 °C). In this case, the temperature gradient and cooling rate in the slurry zone should be much lower than those of the mushy zone, and this can provide the appropriate solidification conditions for the growth of coarse dendrites. Moreover, the large dimension of the slurry zone will ensure that solidification of the floating grains occurs before they are entrapped by the mushy zone. The hot-top mold is commonly used in the DC casting of aluminum alloys, which description and schematic can be found in the work of Nadella et al. [7]. The melt is introduced to the mold through an open inlet during casting. Therefore, the fluid flow below the inlet is gentle during DC casting. However, the sump of the DC-cast is relatively short, thus the buoyancy-induced convection from the periphery to the center of billet (thermal and solutal buoyancy) would be highlighted, as shown in Figure 7. Here, the shrinkage-induced flow was not considered, because it mainly occurs in the mushy zone [39] where the grains cannot freely move due to their interaction with each other. Thereby, the flow field induced by buoyancy shown in Figure 7 is mainly present in the liquid and slurry zone. The vector results indicate that the molten aluminum moves down the coherency isothermal from the periphery to the center of the billet and recirculation can be identified in the liquid zone as a result of the upward fluid flow.

**Figure 8:**
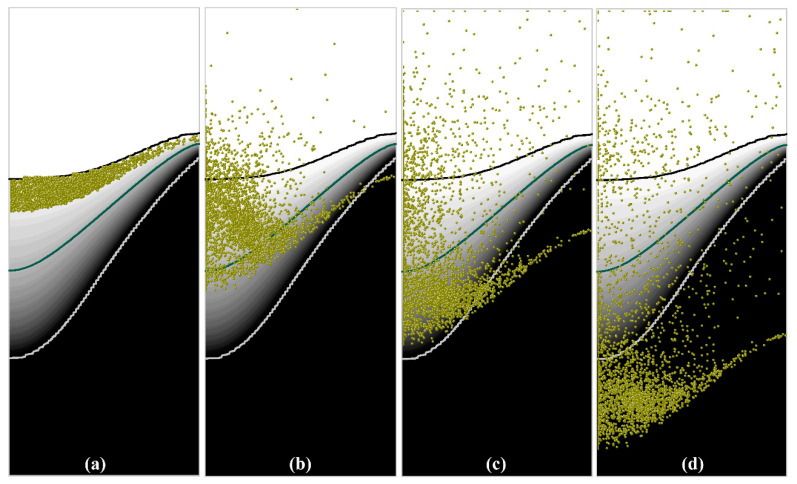
Simulated distribution of the grains in a solidifying DC casting, (**a**) 0 s, (**b**) 10 s, (**c**) 25 s, (**d**) 55 s.

The simulated distribution of the free-floating grains (represented by the particles with a diameter of 100 μm) in a solidifying DC casting is presented in Figure 8 for different calculation times. Figure 8a shows the initial distribution of the grains in DC casting. Under the action of the fluid flow and the forces (see in Table 1), the grains floating in the transition region exhibit various motion behaviors. During DC casting, most of the grains moved toward the center of the billet, and only a few grains settled immediately near the billet surface. After 10 s of simulation, a clearly dispersive grains distribution is obtained in the billet. Driven by the recirculating flow, some of the grains were transported into the liquid zone, where some may completely remelt and the rest will move again into the slurry zone. During this process, some grains floating close to the coherency isotherm were captured by the mushy zone where they can no longer move freely and are confined by the surrounding grain structure until the complete solidification of the remnant liquid, as shown in Figure 8c,d. Meanwhile, most of the grains were transported to the central region of the billet where some of them continue to float and others were entrapped by the mushy zone. An examination of the grain’s distribution shown in Figure 8d indicates that most grains settled in the central region, and only a few grains were found near the surface of the billet.

**Figure 9:**
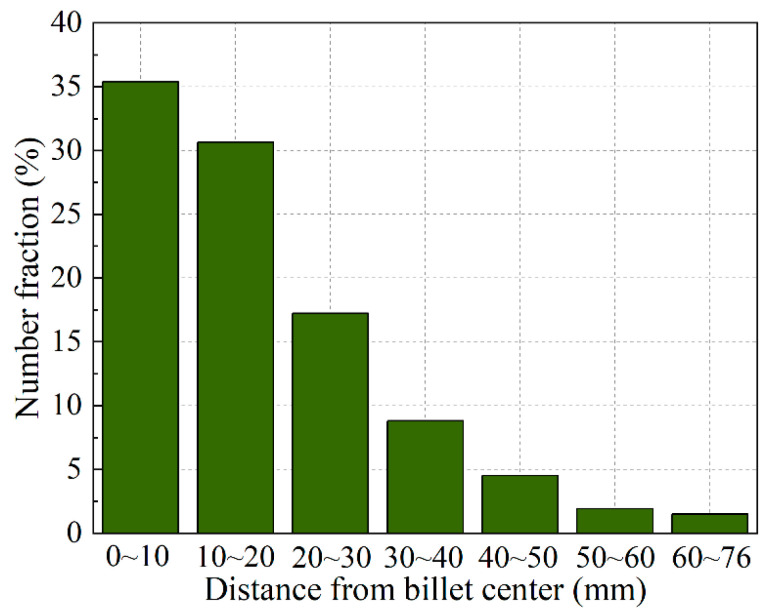
Number fraction of the grains that were entrapped at various distances from the billet center.

Statistical analysis of the distribution of the grains entrapped at various distances from the center of the billet was carried out for the image presented in Figure 8d, and the obtained results are given in Figure 9. Although the grains exhibit different motion behavior in the transition region during casting, a majority of the grains were transported to the central region by the downward convective flow along the coherency isothermal, and then were captured by the mushy zone and settled. Therefore, the largest fraction of the settled grains appears at the center of the billet, and the adjacent region also contains a large number of the grains. Only a few grains were entrapped at the upper quarter and surface of the billet. The distribution of floating grains in the central region of billet is in consistent with the experimental results shown in Figure 6, which also illustrates that the center of the DC-cast billet possesses most floating grains, and the number and area ratio of floating grains decreases away from the billet center. The comparison between the simulated and experimental distribution of floating grains indicates the validity of the numerical model developed in this work. However, we also note a difference in the distribution of floating grains between the simulated and experimental results. The floating grains were only experimentally observed at the central region of the billet, whereas a few grains were also numerically obtained even at the region nearby the billet surface, as shown in Figure 8 and Figure 9. This can be attributed to the formation of the floating grains as described earlier, and only the grains underwent the special freezing conditions in the slurry zone and were then captured by the mushy zone are “floating grains”. However, the simulated results indicate that the grains that freely float in the transition region are in fact not entirely transported to and captured at the central region during DC casting; rather, some of them also settle near the billet surface. However, the slurry zone is very narrow near the billet surface, and the grains settled in this region do not have enough time to grow. Therefore, they settled and solidified along with the surrounding grain structure at the mushy zone. In this case, the grains that settled early near the billet surface are not the “floating grains” because they cannot exhibit the micro-scale features (shown in Figure 5) of the floating grains, so that they will not influence the localized segregation. Thereby, the difference between the simulated and experimental distribution of floating grains is reasonable. In addition, the grains grow slowly while freely floating in the slurry zone. The size of the grains will gradually change, and this should influence their motion and distribution.

**Figure 10:**
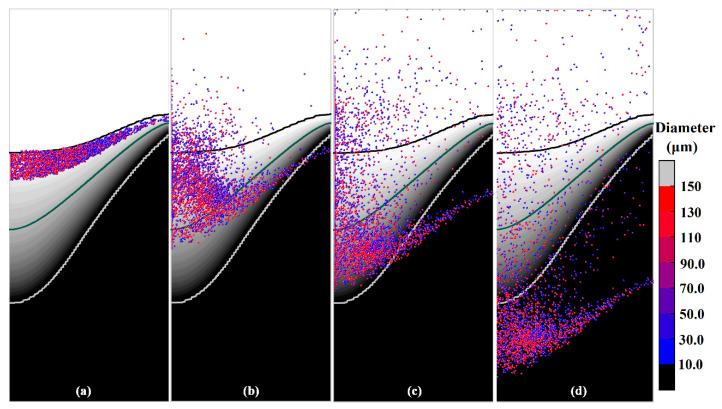
Simulated distribution of the grains with different diameters at different calculation times of (**a**) 0 s, (**b**) 10 s, (**c**) 25 s, (**d**) 55 s.

**Figure 11:**
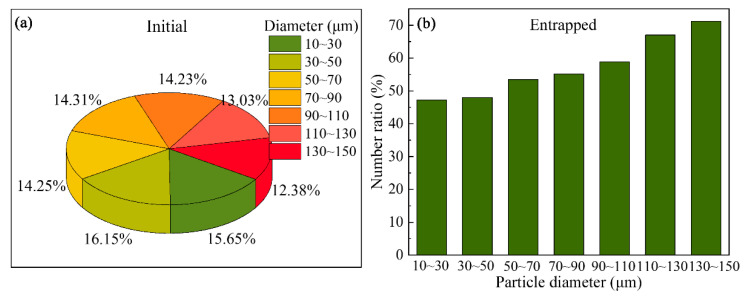
Number ratio of the grains with different diameters (**a**) at 0 s, (**b**) entrapped at 55 s.

To investigate the effect of the grain size on the distribution of the floating grains in a DC-cast billet, another simulation was carried out with grains of different diameters, and the results are shown in Figure 10 and Figure 11. The simulated distributions of the grains with different diameters at different calculation times are presented in Figure 10. At t = 0 s, the grains with different diameters are dispersed in the region with a solid fraction between 0 and 0.1; the corresponding results for the number fraction are shown in Figure 11a. As mentioned above, the grains distribution in Figure 10a is obtained by numerical simulation. Therefore, different number fraction values are obtained for the grains with different sizes, and the grains with the diameters in the 10–30 μm range show the highest fraction. As shown in Figure 10, the grains with different sizes exhibit the same motion behavior in the DC casting, i.e., while some of them moved into the liquid zone, most were transported to the central region of the billet and settled there. However, the grains with relatively larger sizes appear to contribute to sedimentation as can be identified from Figure 10b–d. The statistical data presented in Figure 11b show the number fractions of the grains entrapped in the solid at t = 55 s. The grains with the sizes in the 130–150 μm range make the largest contribution of approximately 70%, while the number fraction of the grains with the size of 10–30 μm in the solid is only approximately 47%. This confirms that the increased size of the grains promotes their sedimentation, and this can be explained according to the particle transport model. The motion of the grains in the recirculating flow is mainly dominated by the drag force due to the relatively large velocity of the molten aluminum, and therefore the grains can be transported to the liquid zone even with a large size. Otherwise, the grains move downward under the action of the gravity. Dividing the equation of the particle transport model (listed in Table 1) by the $d_{p}^{3}$, we find that the increased grain size will reduce the influence of the upward drag force and the Saffman lift force. Therefore, if the grains moved close to the coherency isotherm, their large size would promote their sedimentation.

The findings in this work clearly indicates that the accumulation of the floating grains in DC-cast billets accounts for the occurrence of the centerline negative segregation. And the results of numerical simulation explained the reason for the special distribution of floating grains. The buoyancy-induced convection existing in the slurry zone mainly determines the motion and distribution of the free-floating grains. In this case, the negative centerline segregation can be minimized by preventing the sedimentation of the floating grains at the central region of the billet. This can be achieved by changing the fluid flow during casting, e.g., electromagnetic casting, the simulations can offer the instruction in technical subjects.

## 5. Conclusions

The motion and distribution of the floating grains in DC casting of aluminum alloys were investigated by experiments and numerical modeling. Based on the obtained results, the following conclusions can be drawn.

(1) Negative centerline segregation was typically observed at the central region of the DC-cast billet where some peculiar grains that are twice as large as the regular grains were correspondingly identified.
(2) The peculiar grains consist of the interior coarse- and periphery fine-DAS dendrites, and the coarse-DAS dendrites contain lower concentrations of solutes than the fine-DAS dendrites. Based on their special microscopic features, the peculiar grains observed in the central region of the billet can be confirmed to be the floating grains.
(3) The floating grains contribute the largest share of the grain structure at the billet center, with approximately 16% in the number fraction and nearly 70% in area fraction; these values then decrease gradually to 1.9% and 5.1% respectively, at the distance of 25 mm from the billet center.
(4) The slurry zone accounts for nearly a half of the transition region even though it has a much smaller temperature range compared to the mushy zone. The convection induced by buoyancy consists of the downward and recirculating fluid flow in the slurry and liquid zones.
(5) The grains that float in the transition region exhibit different motion behaviors, i.e., settling to mushy zone, floating in slurry zone, and moving upward to the liquid zone. Most grains were transported to the central region of the billet, and then were captured by the mushy zone and settled. The simulated distribution of the floating grains is consistent with the experimental results.
(6) The increased size of the grains promotes their sedimentation and entrapment.

## Figures and Tables

**Table 1:** Conservation equations for the mathematic model.

Description	Equations	Supplements
Mass conservations	$\nabla \cdot \left(\rho \vec{u}\right)=0$	-
Momentum conservations	$\frac{\partial \left(\rho \vec{u}\right)}{\partial t}+\nabla \cdot \left(\rho \vec{u}\vec{u}\right)=\nabla \cdot \left(\mu_{eff}\nabla \vec{u}\right)-\nabla p+\rho \vec{g}+{\vec{S}}_{u}$	${\vec{S}}_{u}=\rho \vec{g}\beta_{T}\left(T-T_{R}\right)+\frac{{\left(1-f_{l}\right)}^{2}}{f_{l}{}^{3}+\xi }A_{m}\left(\vec{u}-{\vec{u}}_{s}\right)$
Energy conservations	$\frac{\partial \left(\rho H\right)}{\partial t}+\nabla \cdot \left(\rho \vec{u}H\right)=\nabla \cdot \left(k_{T,}{}_{eff}\nabla T\right)$	$H=H_{ref}+\int_{T_{ref}}^{T}c_{p}dT+f_{l}L$
Particle transport model	$\rho_{p}\frac{\pi }{6}d_{p}^{3}\frac{d{\vec{u}}_{p}}{dt}={\vec{F}}_{\mathrm{drag}}+{\vec{F}}_{p}+{\vec{F}}_{b}+{\vec{F}}_{\mathrm{VM}}+{\vec{F}}_{s}$	${\vec{F}}_{\mathrm{drag}}=\frac{\pi }{8}d_{p}^{2}C_{D}\rho_{l}\left|{\vec{u}}_{p}-{\vec{u}}_{l}\right|\left({\vec{u}}_{p}-{\vec{u}}_{l}\right)$ ${\vec{F}}_{p}=-\frac{\pi }{6}d_{p}^{3}\nabla p$ ${\vec{F}}_{b}=\frac{\pi }{6}d_{p}^{3}(\rho_{l}-\rho_{p})\vec{g}$ ${\vec{F}}_{VM}=\frac{\pi }{6}d_{p}^{3}\rho_{l}C_{VM}\frac{d}{dt}\left({\vec{u}}_{l}-{\vec{u}}_{p}\right)$ $\begin{array}{cc} {\vec{F}}_{s}= & 1.61d_{p}^{2}{\left(\rho_{l}\mu_{eff}\right)}^{1/2}{\left|\nabla \times {\vec{u}}_{l}\right|}^{-1/2}\\ & \left(\left({\vec{u}}_{l}-{\vec{u}}_{p}\right)\times \left(\nabla \times {\vec{u}}_{l}\right)\right) \end{array}$

**Table 2:** Operation parameters and thermo-physical parameters.

Parameters	Values
Casting speed, m/min	0.09
Billet diameter, mm	152
Casting temperature, K	970
Density, kg/m^3^	2460
Particle dendity, kg/m^3^	2550
Solid specific heat, J/(kg·K)	958
Liquid specific heat, J/(kg·K)	1054
Liquid thermal conductivity, W/(m·K)	95
Solid thermal conductivity, W/(m·K)	180
Thermal expansion coefficient, K^−1^	1.17 × 10^−4^
Liquid viscosity, Pa·s	0.0013
Latent heat, J/kg	392,000
Melting point of pure aluminum, K	933.5
Liquidus temperature, K	923

## Nomenclature

Nomenclature			
ρ	Density of continuous phase (kg·m^−3^)	$c_{p}$	Specific heat (J·kg^−1^·K^−1^)
$\vec{u}$	Velocity (m·s^−1^)	L	Latent heat (J·kg^−1^)
μ	Viscosity (Pa·s)	$T_{R}$	Reference temperature (K)
t	Time (s)	$\rho_{p}$	Density of particles (grains) (kg·m^−3^)
p	Pressure (Pa)	$d_{p}$	Particle diameter (μm)
$\vec{g}$	Gravity acceleration (m·s^−2^)	${\vec{u}}_{p}$	Particle velocity (m·s^−1^)
${\vec{S}}_{u}$	Momentum source term (kg·m^−2^·s^−2^)	${\vec{F}}_{\mathrm{drag}}$	Particle drag force (N)
$\beta_{T}$	Thermal expansion coefficient (K^−1^)	${\vec{F}}_{p}$	Pressure gradient force (N)
T	Temperature (K)	${\vec{F}}_{b}$	Buoyancy force (N)
ξ	A small positive number	${\vec{F}}_{\mathrm{VM}}$	Virtual mass force (N)
$f_{l}$	Mass fractions of liquid phase	${\vec{F}}_{s}$	Saffman lift force (N)
$A_{m}$	Permeability coefficient (m^−2^)	$T_{surf}$	Temperature of billet surface (K)
H	Enthalpy (J·kg^−1^)	$T_{e}$	Environment temperature (K)
$k_{T}$	Heat conductivity (W·m^−1^·K^−1^))	h	Heat transfer coefficient (W·m^−2^·K^−1^)
$f_{S}$	Solid fraction		
Subscripts			
*ref*	Reference value	*contact*	Mold contact value (2000)
*eff*	Effective value	*air*	Air gap value (150)
